# Parents’ knowledge about their children’s congenital heart disease: an observational study

**DOI:** 10.1093/eurpub/ckac131.457

**Published:** 2022-10-25

**Authors:** M Martinato, S Virgis, E Cerantola, H Ocagli, E Cainelli, L Vedovelli, RI Comoretto, D Azzolina, E Monaco, D Gregori

**Affiliations:** 1 Department of Cardiac Thoracic Vascular Sciences and Public Health, Università di Padova, Padua, Italy; 2 Department of Statistics, Informatics, Applications, Università di Firenze, Firenze, Italy; 3 Department of General Psychology, Università di Padova, Padua, Italy; 4 Department of Sciences of Public Health and Pediatrics, Università di Torino, Turin, Italy; 5 Department of Environmental and Preventive Sciences, Università di Ferrara, Ferrara, Italy

## Abstract

**Introduction:**

Congenital heart diseases (CHD) represent abnormalities of cardiovascular structure or function present at birth. The degree of knowledge of parents of children with CHD determines the quality of care and the quality of life of their children. Several studies have shown that parents’ knowledge is still lacking.

**Objectives:**

This study aims to translate and validate in Italian the Leuven Knowledge Questionnaire for Congenital Heart Disease (LKQCHD) and to assess the knowledge of parents of children with CHD about heart defect, treatments, preventive measures, opportunities for physical activity and reproductive problems of their children.

**Methods:**

Translation and validation of the questionnaire were performed using a multistep method: forward translation, backward translation, and pilot testing. Five experts in CHD were included for the validation of the questionnaire. The sample consisted of fifty-four pairs of parents of children with CHD. Parents were contacted by telephone; knowledge was assessed using an electronic questionnaire.

**Results:**

Five items were found to have an Item Content Validity Index (I-CVI) of 0.6, 2 of 0.5, and 2 of 0.2. The Scale ContentValidity Index (S-CVI) was found to be 0.80. Regarding parental knowledge, the results show that almost all parents are able to correctly state the name of the diagnosis and the description and location of the heart defect. However, parental knowledge has important gaps; in particular, parents are less informed about the most characteristic sign of endocarditis, the possibility of contracting endocarditis more than once in a lifetime, and risk factors. Parental knowledge also seems to be lacking regarding symptoms suggesting worsening health status in their children.

**Conclusions:**

The Italian version of the LKQCHD has proved to be a valid tool to measure the level of knowledge of parents of children with CHD, allowing to identify in which areas it is necessary to improve the education addressed to parents.

**Key messages:**

• Assessing the knowledge of parents of children with CHD allows to improve their education.

• The Italian version of the LKQCHD is a valid tool to measure the level of knowledge of parents of children with CHD.

